# Hand washing practice at critical times and its associated factors among mothers of under five children in Debark town, northwest Ethiopia, 2018

**DOI:** 10.1186/s13052-019-0713-z

**Published:** 2019-09-13

**Authors:** Henok Dagne, Laekemariam Bogale, Muluneh Borcha, Anley Tesfaye, Baye Dagnew

**Affiliations:** 1 Department of Environmental and Occupational Health and Safety, Institute of Public Health, College of Medicine and Health Sciences, University of Gondar, Gondar, Ethiopia; 2 Department of Human Physiology, School of Medicine, College of Medicine and Health Sciences, University of Gondar, Gondar, Ethiopia

**Keywords:** Critical time, Hand washing practice, Mothers of under five children

## Abstract

**Background:**

The burden of communicable diseases within developing countries is mainly influenced by poor personal hygiene practices. Hand washing is considered as most cost effective intervention for reducing health problems such as diarrhoea and acute respiratory tract infections. This study aimed to assess hand washing practice at critical times and identify associated factors among mothers of under five children in Debark town.

**Method:**

A community based cross-sectional study design was carried out from May 1–20, 2018 in Debark town. After selection of participants using simple random sampling, face to face interview was performed by using semi-structured pre-tested questionnaire. Data were entered into EPI Info 7 and exported into SPSS 21 for further analysis. Results were presented by simple frequency, percentage and mean for descriptive variables. Binary logistic regression analysis was used to test the association of dependent and independent factors. Variables with 95% confidence interval and *p* ≤ 0.2 during the univariable binary logistic regression analysis were included in the multivariate logistic regression analysis. At the final model variables with *p* ≤ 0.05 were treated as significantly associated factors of hand washing practice at critical times.

**Results:**

Good hand washing practice at critical times was reported in 52.2% (95% CI: 47.5, 57.2%) of study participants. Desirable attitude [AOR = 3.37, 95% CI (2.03, 5.58)], presence of water for washing hands [AOR = 4.86, 95% CI (1.26, 18.69)] and a good knowledge [AOR = 2.98, 95% CI (1.92, 4.60)] were significantly associated factors with hand washing practice at critical times.

**Conclusion:**

The hand washing practice at critical times of study participants was found to be low. A significant proportion of mothers of under five children have a poor hand washing practice at critical times. It is necessary to increase the access to water and to improve knowledge and attitude of mothers to improve their hand washing practice at critical times.

## Background

Human hands are one of the chief vehicles for transmitting infections especially diarrheal and respiratory diseases which are the leading causes of infant and under-five mortalities in developing countries [1]. Many children acquire respiratory, gastrointestinal and skin infections when hands that are contaminated by pathogens touch their nose, mouth and eyes either by themselves, mothers and/or the caregivers at homes or schools [2]. Regular, appropriate hand washing is therefore one of the best ways of preventing the spread of infections and can save millions of lives annually [3].

Hand washing interrupts the transmission of disease agents which can significantly reduce diarrheal, respiratory, skin infections and trachoma [4]. Effective hand washing has been shown to reduce the incidence and prevalence of diarrheal diseases by preventing transmission of a variety of pathogens [5]. Despite the knowledge of this fact, many of children’s caregivers are still not washing their hands effectively [6].

Hand washing with soap and water is one of the most effective measures against infectious disease [7] which can reduce the incidence and prevalence of these diseases [8]. Hand washing mechanically removes pathogenic agents by rinsing with water thereby reducing the number of microbes on the hands in most situations [9]. As several studies have shown, hand washing and basic hygiene behaviour could minimise the spread of germs and as such prevent diarrhoea, acute respiratory infections such as influenza and skin infections [10].

Diarrhoea is the leading cause of child death in Africa and it is the second leading cause of child death globally [11]. Worldwide, 88% of the cases of diarrhoea are attributable to unsafe water, inadequate sanitation and insufficient hygiene [11]. Because of the above-listed scenarios regarding hand washing practice, this study aimed to assess hand washing practice at critical times and identify determinant factors among mothers of under five children in Debark town, Ethiopia, 2018.

## Methods

### Study design and settings

Community based cross-sectional study was conducted from May 1 to 20, 2018 to assess hand washing practice at critical times and associated factors among mothers in Debark town in North Gondar zone of the Amhara region, northwest Ethiopia. It has a total population of 25,350 people. It is divided in to 3 kebeles (smallest administrative unit). The town has 1 hospital, 1 health center, 2 medium clinics, 4 primary clinic and 6 pharmacies [12].

### Sample size determination

Single population proportion formula was used to determine the sample size with the following assumptions: *p* = 38.7 (approximated to 39) (magnitude of hand washing practice at the critical times from Addis Ababa in 2017 [13]), 95% confidence interval, z = the standard normal tabulated value, and α = level of significance and 5% margin of error (d).

$$ n=\frac{{\left({z}_{\frac{a}{2}}\right)}^2\times p\times \left(1-p\right)}{d^2}=\frac{(1.96)^2\times 0.39\times \left(1-0.39\right)}{(0.05)^2}=366 $$

Considering, 10% non-response rate, the final sample became 402.

### Sampling procedures

Simple random sampling technique was used to select study participants. The study participants were selected from the 3 kebeles using simple random sampling technique. The total number of mothers of under five children in the study area was 4070. The number of participants was allocated proportionally based on the number of mothers of under- five at each Kebele. Data collectors went house to house and collect the data through walk through approach randomly. The names and locations of mothers of under-five were found from health extension workers’ log book.

### Data collection procedures

A semi-structured, pre-tested questionnaire adapted from different literature was used to collect data (Additional file 1). Two graduating class Environmental Health students were involved in the data collection process. Training was given for the data collectors regarding the data collection tool, techniques of interview and selection of study participants using simple random sampling. The data collectors visited all selected households and interviewed selected mothers of under-five children. The interview was supervised by the primary investigator. The collected data were checked by the data collectors immediately after finalising the questionnaire before they left the house. Data completeness, quality, and consistency were checked daily.

### Operational definition

#### Hand washing practice at the critical times

Respondents were asked 16 practice questions (Cronbach’s alpha 0.80) whether they wash their hands with water and soap with a 4-scale Likert (1-always, 2-usually, 3-sometimes and 4-never) after toilet visit, before eating, after eating, before and after food preparation, before breastfeeding, after handling babies’ faeces, changing babies’ diapers, after touching money, after touching skin, after patient care, after touching raw food, after touching work cloth, after handling garbage, after handling liquid waste, after sneezing and coughing, before handling raw food and before serving food. The responses forwarded by study participants were dichotomised as 1 for always and usually and 0 for sometimes and never. The responses were added, and the mean was computed. Those participants who scored mean and above mean of the practice questions were considered as having good self-reported hand washing practice.

#### Knowledge about hand washing practice at critical times

Respondents were asked 14 knowledge questions (Cronbach’s alpha 0.82) about their knowledge of critical times of hand washing, whether the respondents ever heard about hand washing at critical times, importance of hand washing at critical times, whether the respondents know that hand washing at critical times reduces gastrointestinal diseases, the disease transmitted by not washing hands, recommended length of time for washing hands, whether they know that hand can transfer disease-causing microorganisms, the role of proper hand washing for prevention of infectious disease such as respiratory infection, knowledge of role of mothers in their children’s hand hygiene, whether they know that long nails can harbour and easily spread bacteria, whether the respondents know that they have to wash their hands after handling babies’ stools, knowledge of hand washing before feeding child, after touching money, after handling garbage, after sneezing and coughing and after defecation. The correct answers were coded as 1 and the wrong answers as 0. Those study participants who scored mean and above mean of the sum of the knowledge questions were considered as having good knowledge.

#### Attitude about hand washing practice at critical times

Respondents were asked about their views for 14 attitudinal questions (Cronbach’s alpha 0.78) with a 4-scale Likert (1-strongly agree, 2-agree, 3-disagree and 4-strongly disagree) dichotomised as desirable and none desirable attitude. Those study participants who scored mean and above mean of the attitude questions were considered as holding the desirable attitude.

### Data management and statistical analysis

Data were entered using Epi-info version 7 and exported into SPSS version 21 for further analysis. For most variables, data were presented by frequencies and percentages. Binary logistic regression analysis was used to choose variables for the multivariable binary logistic regression analysis. Variables with *p* ≤ 0.2 during univariable binary logistic regression analysis were entered into multivariable binary logistic regression analysis for controlling the possible effect of confounders. Finally, variables which had significant association with hand washing practice at critical times were identified on the basis of AOR with 95% CI and *p* ≤ 0.05. Hosmer and Lemeshow goodness- of -fit test was used to check model fitness.

## Results

### Sociodemographic characteristics of study participants

Four hundred and two (402) study participants were included in this study with a 100% response rate. Majority (79.6%) of the study participants were married. Two hundred and forty-five (60.9%) study participants were housewives. Only 81 (20.1%) of the respondents had completed diploma and above educational status. Three hundred and thirty-eight (84.1%) of the study participants had heard about hand washing at critical times. Almost all (95.5%) of the study participants reported that they have sufficient water for hand washing (Table 1).

**Table 1:** Sociodemographic profiles of study participants Debark town, 2018 (*n* = 402)

Variables	Frequency (*n*)	Percent (%)
Age in years		
20–28	134	33.3
29–32	69	17.2
33–40	105	26.1
41–70	94	23.4
Marital status		
Currently married	319	79.6
Not married	83	20.4
Religion		
Orthodox	330	82.1
Muslim	72	17.9
Educational status		
No education	110	27.4
Primary school	150	37.3
Secondary school	61	15.2
College and above	81	20.1
Occupation		
Housewife	245	60.9
Employed	157	39.1
Family size		
< 5	292	72.6
≥ 5	110	27.4
Ever heard about hand washing at critical times		
Yes	338	84.1
No	64	15.9
Frequency of visit by HEW		
Visited at least once per month	320	79.6
Never visited in a month	82	20.4
Self-reported knowledge of components of HEP		
Yes	277	68.9
No	125	31.1
Self-reported availability of water for hand wash		
Yes	384	95.5
No	18	4.5
Source of water		
Protected	368	91.5
Unprotected	34	8.5
Type of hand washing facility		
Water only	65	16.2
Water and soap/ash	337	83.8

*HEW* Health Extension Worker, *HEP* Health Extension Program

### Knowledge, attitude and self-reported practice of hand washing at critical times

**Fig. 1:**
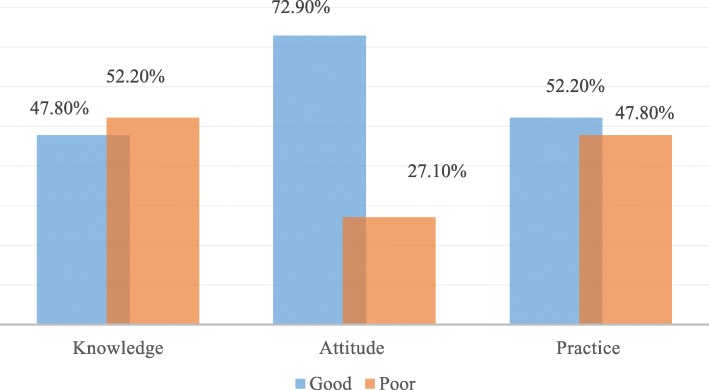
Knowledge, attitude and practice of mothers about hand washing at critical times (*n* = 402)

Only 52.2% (95% C. I (47.5, 57.2%)) of the study participants had good self-reported hand washing practice at critical times. A majority (72.9%) of respondents had a desirable attitude (Fig. 1).

### Factors associated with self-reported hand washing practice at critical times

Information about hand washing, knowledge of the component of HEP, availability of water, the type of hand washing material, knowledge regarding and attitude about hand washing at the critical times were variables with *p*-value ≤0.2. Only knowledge, attitude and water availability were significantly associated with hand washing practice at critical times.

Study participants who reported having sufficient water for hand washing were more likely to report better hand washing practice than those who reported a lack of water with estimated odds ratio of 4.86 (AOR = 4.86, 95% CI (1.27,18.69)).

Knowledgeable mothers were more likely to have higher self-reported hand washing practice than those with poor knowledge about hand washing at critical times with estimated odds ratio of 2.98 (AOR = 2.98, 95% CI (1.92, 4.60)).

Study participants with the desirable attitude were more likely to have self-reported hand washing practice as compared to those with undesirable attitude with estimated odds ratio of 3.37 (AOR = 3.37, 95% CI (2.03,5.58)) (Table 2).

**Table 2:** Associated factors of self-reported hand washing practice at critical times among mothers of under five children at Debark town, 2018 (*n* = 402)

Variables/ Categories	Hand washing practice		COR (95% C.I.)	AOR (95% C.I.)
	Good (%)	Poor (%)		
Ever heard about hand washing at critical times				
Yes	182 (53.8)	156 (46.2)	1.5 (0.88,2.57)	–
No	28 (43.8)	36 (56.3)	1	
Self-reported knowledge of components of HEP				
Yes	163 (58.8)	114 (41.2)	2.37 (1.54, 3.66)	1.51 (0.94,2.45)
No	47 (37.6)	78 (62.4)	1	
Self-reported availability of water for hand washing				
Yes	207 (53.9)	177 (46.1)	5.85 (1.67, 20.53)	4.86 (1.27,18.69)*
No	3 (16.7)	15 (83.3)	1	1
Type of hand washing material				
Water only	25 (38.5)	40 (61.5)	1	1
Water and soap/ash	185 (54.9)	152 (45.1)	1.95 (1.13,3.35)	–
Knowledge				
Good	130 (67.7)	62 (32.3)	3.41 (2.26,5.14)	2.98 (1.92,4.60)***
Poor	80 (38.1)	130 (61.9)	1	1
Attitude				
Desirable	178 (60.8)	115 (39.2)	3.72 (2.32,5.98)	3.37 (2.03,5.58)***
Undesirable	32 (29.4)	77 (70.6)	1	1

*p ≤ 0.05, ***p ≤ 0.001, *CI* confidence interval, AOR, COR Hosmer and Lemshow goodness-of-fit

## Discussion

In the current study two hundred ten (52.2%) with 95% C. I (47.5, 57.2%) of the study subjects reported that they had good hand washing practice at the critical times. This is in line with previous reports among caregivers of under-five children in a rural Nigeria [14]. The hand washing practice at critical times in the current study was lower than a study conducted among mothers in Mandalay [15] and among mothers of children 0–59 months of age in Lagos, Nigeria [16]. However, it was higher than the hand washing practice reported among mothers of under-five children in Wondogenet [17], Bandung, Indonesia [18], in Addis Ababa, Ethiopia [13].

The differences in the practice of hand washing among mothers may be because of differences in socioeconomic status, level of health services, educational status, living standard difference among the study settings and the difference in the tool used for assessing hand washing practice and techniques of assessment. For example, the method used in the current study and in studies [13, 18] used self-reported practice whereas the study in Wondogenet [17] was supported by observation. Self-reported hand-washing rates are inflated when compared to observed data, meaning that good or desirable behaviour is self-reported more frequently than it is observed [19]. The proportion of mothers with good hand washing practice in the current study is higher than a study conducted in Kirkos, Addis Ababa. This might be due to the fact that the study done in Kirkos was among one of the most slum area of the City [13].

In the current study knowledge, attitude and water availability were significantly associated with hand washing practice of mothers at critical times. Mothers with a good knowledge were tended to have better self-reported hand washing practice than those with poor knowledge. This was in line with previous studies [17, 20–24]. But in other studies [14, 25–30] knowledge was not associated with hand hygiene practice. Surface level knowledge do not lead to desirable behavioural change that elicits to better practice.

Mothers with a desirable attitude had better self-reported practice. This is in line with other studies [22, 27]. However, attitude was not associated with hand washing practice in other studies [25, 31, 32]. This might be because of other potential factors which will contribute to the practice than attitude in earlier studies. Study subjects who reported that they have sufficient water for hand washing had better self-reported hand washing practice at critical times. Water availability is a significant factor in hand washing practice in earlier studies, too [14, 26, 33].

## Conclusion

Hand washing practice at critical times is important to reduce diarrheal diseases and respiratory infection. The practice among mothers was relatively low. The availability of water, knowledge and attitude were significant factors affecting hand washing practice. Therefore, increasing the availability of water and devising policies and programs targeted at improving the knowledge and attitude of mothers regarding hand washing practice at critical times is crucial to reduce under-five diarrheal disease and respiratory infection.

### Limitations of the study

The use of relative scale than global sum score in analyzing the response variable in the current study made the comparison with earlier studies difficult. Because of the self-reported nature of the study, recall bias and social desirability bias were also limitations. Besides, cause-effect relationship can not be seen as this is cross sectional study.

## Additional file

**Additional file 1:** English version questionnaires. University of Gondar College of Medicine and Health Science, Institute of Public Health Department of Environmental and Occupational health and Safety Structured questionnaire regarding Hand washing practice at critical times and its associated factors among mothers of under five children in Debark town, northwest Ethiopia, 2018.

## Data Availability

The dataset is accessible at the corresponding author upon request.

## Abbreviations

AOR: Adjusted Odds Ratio
CI: Confidence Interval
COR: Crude Odds Ratio
EPI Info: Epidemiological Information
HEP: Health Extension Program
SPSS: Statistical Package for Social Sciences
