# Supplementary material for: Hand washing practice at critical times and its associated factors among mothers of under five children in Debark town, northwest Ethiopia, 2018
Source: Ital J Pediatr. 2019 Sep 13;45:120. doi: 10.1186/s13052-019-0713-z (PMC6743165; doi:10.1186/s13052-019-0713-z)
Supplement: Supplementary file 1 — Additional file 1: English version questionnaires. University of Gondar College of Medicine and Health Science, Institute of Public Health Department of Environmental and Occupational health and Safety Structured questionnaire regarding Hand washing practice at critical times and its associated factors among mothers of under five children in Debark town, northwest Ethiopia, 2018. [file 13052_2019_713_MOESM1_ESM.docx]

**English version questionnaires**

**University of Gondar College of Medicine and Health Science, Institute of Public Health Department of Environmental and Occupational health and Safety Structured questionnaire regarding Hand washing practice at critical times and its associated factors among mothers of under five children in Debark town, northwest Ethiopia, 2018**

| **S.no** | **Part 1 ፡Socio Demographic factors** | | | **skip** |
| --- | --- | --- | --- | --- |
| SD101 | Age in years | | ________years |  |
| SD102 | Marital status? | | 1. Currently married 2. Currently not married |  |
| SD103 | What is your religion? | | 1.Orthodox  2.Muslim  5. Others specify------- |  |
| SD104 | What is your educational level? | | 1.unable to read and write  2.primary level(1-8)  3.secondary level(9-12)  4.college level and above |  |
| SD105 | What is your work responsibility | | 1.House wife  2. Employed |  |
| SD106 | What is your family size? | | ____________ |  |
| SD107 | Have you ever heard about hand washing at critical times? | | 1.Yes  2.No |  |
| SD108 | If “yes” to SD 107, what was your source of information regarding hand washing at critical times? | | 1.television  2. radio  3.health extension workers  4. family  5. teachers  6. others (specify)_______ |  |
| SD109 | Have you ever been visited by health extension workers? | | 1.Yes  2.No |  |
| SD110 | How often do the health extension workers visit you? | | 1. at least once per month  2.Never visited within a month |  |
| SD111 | Do you know components of health extension program? | | 1.Yes  2.No |  |
| SD112 | Do you have sufficient water for hand washing? | | 1.Yes  2.No |  |
| SD113 | What is your water source? | | 1.protected water source  2.unprotected water source |  |
| SD114 | What is the type of hand washing facility you usually use? | | 1.water only  2.water and soap  3.water and ash |  |
| SD115 | Where is the location of your hand washing facility? | | 1. near to latrine  2. near to household  3. there is no separate place |  |
| **Part 2: Knowledge on hand washing practice at critical times** | | | |  |
| K201 | Have you ever heard about critical times of hand washing | 1.Yes  2.No | |  |
| K202 | What does hand washing practice at critical times includes?  (Circle all that applies) | 1. before eating  2. after defecation  3. after cleaning child’s stool  4. before cooking  5. After touching money  6. after sneezing and coughing  7.after handling garbage  8.after handling raw food  9.after caring care of patients with diarrhea  10.after touching working clothes  11.before feeding child  12. others(specify)________ | |  |
| K203 | What is the importance of hand washing at critical times? | 1. Helps prevent diseases  2.Helps protect your children from infections  3. Helps limits spread of infections in the community  4. Others (specify…)  5. do not know | |  |
| K204 | Hand washing at critical times may help to reduce gastrointestinal disease? | 1.Yes  2.No  3.I don’t know | |  |
| K205 | What diseases can be contracted by not washing hands? | 1. Malaria  2. Typhoid  3. Diarrheal Disease  4. Others (specify…)  5. do not know | |  |
| K206 | Hands need to be washed at least for how long? | 1._____minutes/____seconds  2. I don’t know | |  |
| K207 | Hands not washed properly can transfer disease-causing microorganisms | 1.Yes  2.No  3.I don’t know | |  |
| K208 | Proper hand washing at critical times can help prevent infectious disease such as respiratory infection | 1.Yes  2.No  3.I don’t know | |  |
| K209. | Mothers have a significant role in the hand hygiene of their children | 1.Yes  2.No  3.I don’t know | |  |
| K210 | Long nails can harbor and easily transfer disease causing microorganisms | 1.Yes  2.No  3.I don’t know | |  |
| K211 | Mothers are not expected to wash their hands after handling baby’s stools. | 1.Yes  2.No  3.I don’t know | |  |
| K212 | Mothers are expected to wash their hands before feeding their child. | 1.Yes  2.No  3.I don’t know | |  |
| K213. | It is mandatory to wash hands after touching money. | 1.Yes  2.No  3.I don’t know | |  |
| K214. | Mothers should wash their hands after handling garbage | 1.Yes  2.No  3.I don’t know | |  |
| K215. | Washing hands with soap and water after toilet visit is essential for preventing gastrointestinal disease. | 1.Yes  2.No  3.I don’t know | |  |
| K216. | Washing hands after sneezing and coughing on hands is essential. | 1.Yes  2.No  3.I don’t know | |  |
| **Part 3 : Attitude questions** | | | |  |
| A301 | Washing hands after toilet visit prevent disease | 1.Strongly agree  2. Agree  3.Disagree  4. Strongly disagree | |  |
| A302 | Mothers need to wash hands with soap and water after toilet visit | 1.Strongly agree  2. Agree  3.Disagree  4. Strongly disagree | |  |
| A303 | Mothers need to wash hands after and before eating with soap and water. | 1.Strongly agree  2. Agree  3.Disagree  4. Strongly disagree | |  |
| A304 | Nail cleanliness is important to prevent foodborne diseases | 1.Strongly agree  2. Agree  3.Disagree  4. Strongly disagree | |  |
| A305 | Using hand towels after washing hands will reduce bacterial load on hands | 1.Strongly agree  2. Agree  3.Disagree  4. Strongly disagree | |  |
| A306. | It is the responsibility of mothers to teach their children how to wash hands | 1.Strongly agree  2. Agree  3.Disagree  4. Strongly disagree | |  |
| A307. | Knowledge on proper hand washing procedure and critical times helps for better hand washing practice. | 1.Strongly agree  2. Agree  3.Disagree  4. Strongly disagree | |  |
| A308. | Washing my hands at critical times is my number one priority. | 1.Strongly agree  2. Agree  3.Disagree  4. Strongly disagree | |  |
| A309. | Washing my hands at critical times using standard procedures is my habitual action | 1.Strongly agree  2. Agree  3.Disagree  4. Strongly disagree | |  |
| A310. | I believe frequent and appropriate hand washing can reduce disease burden of my family. | 1.Strongly agree  2. Agree  3.Disagree  4. Strongly disagree | |  |
| A311. | Absence of soap is sufficient reason for not washing my hands. | 1.Strongly agree  2. Agree  3.Disagree  4. Strongly disagree | |  |
| A312. | Proper hand washing at critical times helps to decrease diarrheal disease and acute respiratory infection | 1.Strongly agree  2. Agree  3.Disagree  4. Strongly disagree | |  |
| A313. | Health education helps to improve hand hygiene compliance of family members. | 1.Strongly agree  2. Agree  3.Disagree  4. Strongly disagree | |  |
| A314. | Noncompliance to hand hygiene at critical times results in risk of diarrheal diseases | 1.Strongly agree  2. Agree  3.Disagree  4. Strongly disagree | |  |
| **Part 4: Practice questions** | | | |  |
| P401. | How often do you wash hands with water and soap after sneezing and coughing? | 1.Always  2. Usually  3. some times  4.Never | |  |
| P402. | How often do you wash hands with water and soap before eating? | 1.Always  2. Usually  3. some times  4.Never | |  |
| P403 | How often do you wash hands with water and soap after eating? | 1.Always  2. Usually  3. some times  4.Never | |  |
| P404 | How often do you wash hands with water and soap before food preparation? | 1.Always  2. Usually  3. some times  4.Never | |  |
| P405 | How often do you wash hands with water and soap before breastfeeding? | 1.Always  2. Usually  3. some times  4.Never | |  |
| P405 | How often do you wash hands with water and soap after handling babies’ excreta? | 1.Always  2. Usually  3. some times  4.Never | |  |
| P406 | How often do you wash hands with water and soap after touching money? | 1.Always  2. Usually  3. some times  4.Never | |  |
| P407 | How often do you wash hands with water and soap after touching skin? | 1.Always  2. Usually  3. some times  4.Never | |  |
| P408 | How often do you wash hands with water and soap after patient care? | 1.Always  2. Usually  3. some times  4.Never | |  |
| P409 | How often do you wash hands with water and soap after touching raw food? | 1.Always  2. Usually  3. some times  4.Never | |  |
| P410 | How often do you wash hands with water and soap after touching work cloth? | 1.Always  2. Usually  3. some times  4.Never | |  |
| P411 | How often do you wash hands with water and soap after handling garbage? | 1.Always  2. Usually  3. some times  4.Never | |  |
| P412 | How often do you wash hands with water and soap before handling raw food? | 1.Always  2. Usually  3. some times  4.Never | |  |
| P413 | How often do you wash hands with water and soap before serving food? | 1.Always  2. Usually  3. some times  4.Never | |  |
| P414 | How often do you wash hands with water and soap after toilet visit? | 1.Always  2. Usually  3. some times  4.Never | |  |
| P415 | How often do you wash hands with water and soap after handling liquid waste? | 1.Always  2. Usually  3. some times  4.Never | |  |
| P416 | How often do you wash hands with water and soap after changing baby’s diapers? | 1.Always  2. Usually  3. some times  4.Never | |  |
